# A Scoping Review to Assess Sexual and Reproductive Health Outcomes, Challenges and Recommendations in the Context of Climate Migration

**DOI:** 10.3389/fgwh.2021.757153

**Published:** 2021-10-15

**Authors:** Kim Robin van Daalen, Sara Dada, Rita Issa, Maisoon Chowdhury, Laura Jung, Lucy Singh, Diarmuid Stokes, Miriam Orcutt, Neha S. Singh

**Affiliations:** ^1^Cardiovascular Epidemiology Unit, Department of Public Health and Primary Care, Cambridge University, Cambridge, United Kingdom; ^2^UCD Centre for Interdisciplinary Research, Education and Innovation in Health Systems, School of Nursing, Midwifery and Health Systems, University College Dublin, Dublin, Ireland; ^3^Institute for Global Health, University College London, London, United Kingdom; ^4^Women in Global Health, Washington, DC, United States; ^5^Faculty of Medicine, Leipzig University, Leipzig, Germany; ^6^EGA Institute for Women's Health, University College London, London, United Kingdom; ^7^UCD Library, University College Dublin, Dublin, Ireland; ^8^Health in Humanitarian Crises Centre, London School of Tropical Hygiene and Medicine, London, United Kingdom

**Keywords:** climate migration, sexual health, reproductive health, climate change, scoping review

## Abstract

**Background:** As growing numbers of people may be forced to migrate due to climate change and variability, it is important to consider the disparate impacts on health for vulnerable populations, including sexual and reproductive health (SRH). This scoping review aims to explore the relationship between climate migration and SRH.

**Methods:** We searched PubMed/MEDLINE, CINAHL Plus, EMBASE, Web of Science, Scopus, Global Health and Google for peer-reviewed and gray literature published before 2nd July 2021 in English that reported on SRH in the context of climate migration. Data were extracted using a piloted extraction tool and findings are reported in a narrative synthesis.

**Results:** We screened 1,607 documents. Ten full-text publications were included for analysis: five peer-reviewed articles and five gray literature documents. Reported SRH outcomes focused on maternal health, access to family planning and antiretroviral therapy, sexual and gender-based violence, transactional sex, and early/forced marriage. Recommendations to improve SRH in the context of climate migration called for gender-transformative health systems, education and behavior change programmes, and the involvement of local women in policy planning and programme implementation.

**Discussion:** While the disparate impacts of climate change and migration are well-established, primary data on the scope of impact due to climate migration is limited. The SRH outcomes reported in the literature focus on a relatively narrow range of SRH domains, emphasizing women and girls, over men. Achieving holistic and equitable SRH in the context of climate migration requires engaging all genders across the range of SRH outcomes and migration contexts. This review highlights the need for further empirical evidence on the effect of climate migration on SRH, with research that is context-specific and engages communities in order to reflect the heterogeneity of outcomes and impact in the climate-migration-SRH nexus.

## Introduction

Beyond environmental and direct health consequences, climate change impacts the patterns and scale of forced human migration through its interactions with social, political, economic, and demographic drivers (1). Between 25 million and 1 billion people are projected to migrate within and across borders by 2050 due to climate change, (2) with 80% of this displaced population estimated to be women and girls (3).

Worldwide, women and girls face increased climate-induced health risks compared to men and boys. This is mainly a result from greater exposure and susceptibility to natural hazards and climate-related damage as well as less ability to respond to health impacts, due to cultural and societal norms, including in dimensions of sexual and reproductive health (SRH) (4). For example, the likelihood of adverse pregnancy outcomes (e.g., low birthweight, or miscarriage) is increased by heatwaves, new infectious disease patterns, malnutrition and air pollution resulting from fossil fuel burning (5–7). Furthermore, climate-related disasters can reduce availability and access to reproductive and maternity health services.

Likewise, women and girls experience unique SRH challenges during migration. SRH services are often disrupted or limited by migration, resulting in high levels of unmet needs for contraception, unplanned pregnancies and unsafe abortion, particularly in transit contexts. (8–10) Reduced access to antenatal and postnatal care can ultimately result in higher levels of maternal and neonatal morbidity and mortality. (11, 12) Furthermore, combined stressors from the migration process can contribute to diminished mental health, such as increasing the risk for postnatal depression (13). In addition to these risks, migration is also associated with increased sexual and gender-based violence (SGBV) (8, 11).

However, while there is a significant body of research exploring the interaction between climate change and SRH, as well as between migration and SRH, little is known about specific SRH needs and challenges in situations of climate migration. This is a particular concern due to the potential “risk-multiplier” effect of compounding vulnerabilities of climate change and migration, and a challenge due to the lack of research and operational consensus in defining climate migration. Recommendations on gender-sensitive adaptation for SRH care after both slow-onset and emergency climate disasters are therefore limited (14). This scoping review aims to explore the impact of climate-driven migration on SRH by i) identifying the current knowledge on SRH in the context of climate migration, ii) identifying knowledge gaps for SRH relating to climate migration and iii) highlighting potential drivers and factors of SRH challenges in these contexts as well as opportunities for interventions.

## Methods

A scoping review was conducted in order to examine the range of literature in the field as well as the existing gaps in research, in accordance with the framework described by Arksey and O'Malley (13, 15). This review was prospectively registered on the Open Science Framework (DOI 10.17605/OSF.IO/VYC6K). Findings were reported following the Preferred Reporting Items for Systematic Reviews and Meta-Analyses extension for Scoping Reviews (PRISMA-ScR) guidelines (Supplementary Table 6) (16).

### Definitions

For the purposes of this study, SRH is defined as the dimensions required for women and girls to achieve SRH, including: access to contraception; safe abortion; prevention, screening, diagnosis and treatment of sexually transmitted infections (STIs) including HIV and AIDS; pregnancy-related care including antenatal care, safe delivery and postnatal care; menstrual hygiene; prevention, detection and management of gynaecological conditions and cancers including cervical cancer; and other relevant dimensions including prevention of GBV and child or forced marriage (17, 18). Climate migration is defined as the movement of a person or groups of persons who are obliged or choose to leave their habitual place of residence either temporarily or permanently predominantly for reasons of sudden or progressive change in the environment due to climate change (19).

### Search Methods and Information Sources

Searches were conducted on six electronic databases without time or language restriction through 2nd July 2021: PubMed, CINAHL, EMBASE, Web of Science, Scopus and Global Health. We used search terms for the concepts “climate change,” “migration,” “reproductive health” and “sexual health” which were informed by previous reviews (1, 20, 21). These search terms were supplemented by relevant thesaurus terms from the databases listed above. The full search strategy is provided in Supplementary Table 1. Forward and backward screening of all included full-text and relevant publications to find any additional studies fitting the inclusion criteria was utilised. We searched for grey literature in OpenGrey and Google. Hand-searches of the websites of nine organisations working on SRH in a climate migration context, including the World Health Organization, the United Nations Population Fund, and the Climate and Migration Coalition (full list in Supplementary Table 2).

### Screening Process

All records were imported to EndNote. After removing duplicates, titles and abstracts were uploaded to the software Rayyan (https://rayyan.ai/reviews) and screened independently by two researchers, according to the selection criteria. In the second stage, studies that satisfied the inclusion criteria were screened by full text. Conflicts were resolved among four authors until consensus was reached. Included articles had to meet both components of the following inclusion criteria: i) a primary quantitative, qualitative and mixed-method research article or published comment/editorial and reports; and ii) the article described or investigated SRH in the context of climate migration. Documents were excluded studies if they were i) secondary studies (e.g. reviews), ii) conference proceedings, abstracts, or posters iii) articles that lacked access to the full-text, and iv) articles that did not describe or investigate SRH in the context of climate migration.

### Data Extraction and Critical Appraisal

A predefined data extraction form piloted on one article was subsequently used to extract data from all papers included in the review. The following information was extracted: author, year of publication, geographic location and population characteristics, study methods, and SRH outcome, key findings, challenges, and recommendations. We categorized articles as documents that explicitly investigated SRH in the context of climate migration (i.e. studies that focused on climate migration and an SRH outcome as a main objective) and those that only briefly mentioned this interaction. Each document was extracted by one reviewer and checked by a second reviewer to verify data and ensure its completeness. All included peer-reviewed articles were assessed using the Joanna Briggs Institute (JBI) critical appraisal tool to explore methodological quality of the synthesized knowledge (22). Grey literature was appraised using the Authority, Accuracy, Coverage, Objectivity, Date, Significance (AACODS) checklist (23).

### Data Synthesis

Due to heterogeneity regarding outcome measurement and statistical analysis, we used a narrative synthesis to analyse and report data.

### Patient Involvement

Due to the nature of the study (scoping review), no patients were involved in conceptualising or conducting the study.

## Results

**Figure 1:**
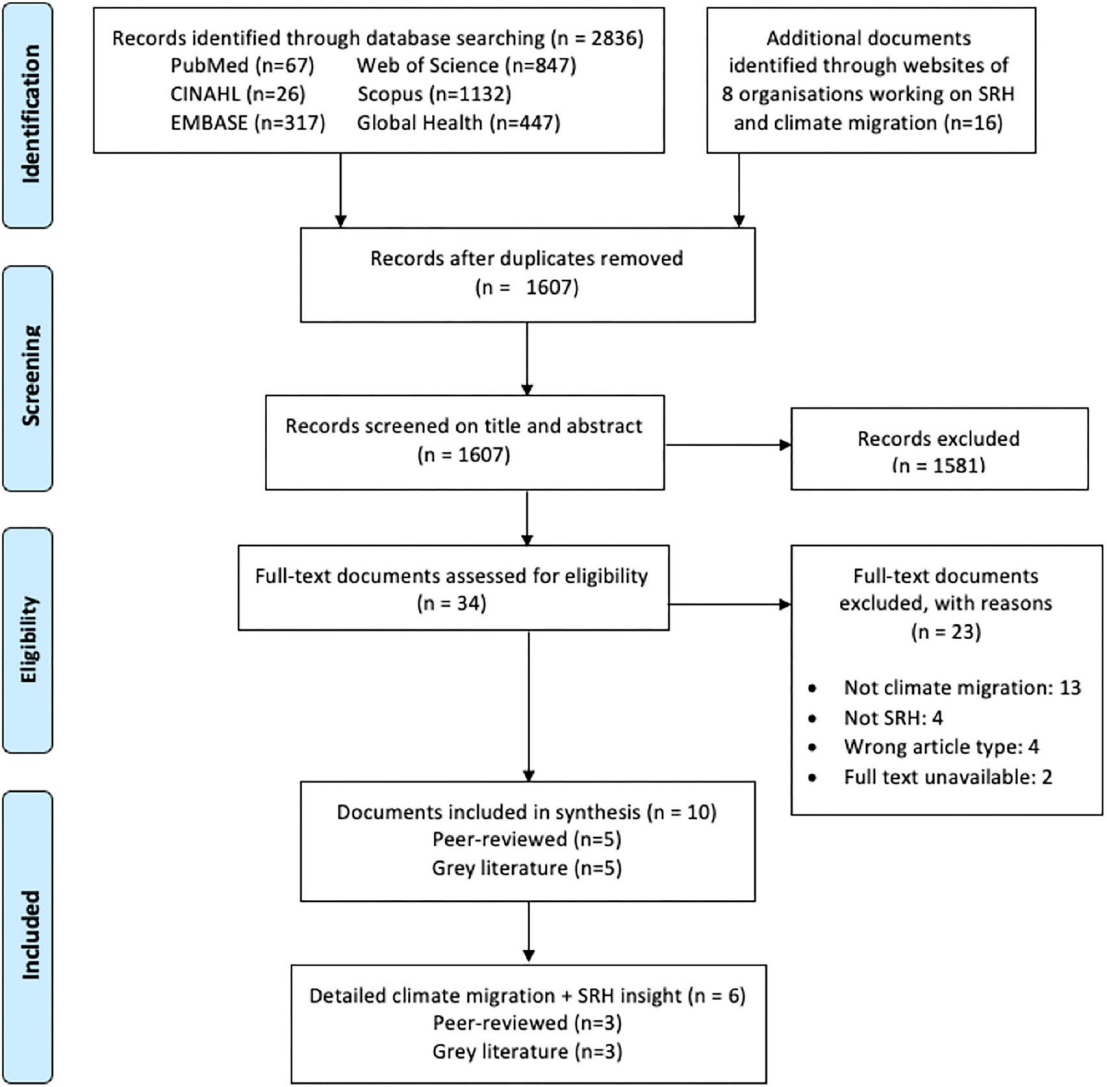
Flow diagram of study selection process.

We identified 2,836 peer-reviewed records and 16 grey literature documents. After removing duplicates, 1,607 articles were screened by title and abstract, followed by 18 articles screened at the full-text stage. In total, 10 articles were included in the review, comprising of five peer-reviewed articles (12, 24–27) and five grey literature articles (29–33). Included articles varied in the level of depth at which they investigated SRH in the context of climate migration, with five of the 10 included articles containing more detailed insight on climate migration and SRH (Figure 1) (6).

### Quality Assessment of Individual Studies

Although the qualitative studies demonstrated reasonable methodological quality, none of them reported ethical approval or reported on the influence of the researcher on the research (Supplementary Table 3) (24, 25). The cross-sectional studies were of lower quality in part due to suboptimal measurements of the exposure and outcome (i.e. self-reporting) and a lack of clarity around the identification and inclusion of confounding variables (Supplement Table 4) (12, 27). Four of the five grey literature articles were of high quality: compiled by a reputable organisation, accurate, objective, timely, and meaningful (29–32). Accuracy, coverage, objectivity and timeliness could not be verified for the final grey literature article - a blog post (Supplementary Table 5) (33). None of the articles were excluded from the review synthesis based on their appraised quality.

### Characteristics of Included Publications

Table 1 reports the characteristics of the ten included articles. The five grey literature articles included reports (*n* = 3), (29–31) a position paper (32), and a blog post (33). The five peer-reviewed studies included qualitative studies using in-depth interviews (*n* = 3) (24), focus group discussions (*n* = 1) (24–26), and quantitative studies using cross-sectional surveys (*n* = 2) (12, 27). Three publications focused on a global or general context, (30–33) whereas the remaining seven publications focused on the African region (*n* = 1) (29), the Asia-Pacific region (*n* = 1) (32), Bangladesh (*n* = 2) (12, 27), Ghana (*n* = 1) (24), Somalia (*n* = 1) (25), Kenya (*n* = 1) (25), Ethiopia (*n* = 1) (25), and India (*n* = 1) (26). The majority of articles focused on SRH outcomes for women and girls (*n* = 9), with only one document including the consideration of LGBTQ+ individuals as a vulnerable population (33). The included articles discussed a range of drivers for climate migration including drought, (25, 26, 29) floods, (12, 27) riverbank erosion, (12, 27) sea-level rise, (33) heatwaves, (33) and general climate variability and change (24, 29–32). The documents discussed a range of SRH components including gender-based violence (*n* = 6), (25, 29–33) maternal and neonatal health (*n* = 5), (12, 25–27, 32) accessibility of SRH services (*n* = 4), (24, 29, 32, 33) and early/forced or child marriages (*n* = 3) (29, 31, 32).

**Table 1:** Summary characteristics of included documents.

**Study**	**Significant level insight**	**Article and/or study type**	**Study setting**	**Climate change related effect discussed**	**SRH outcome**	**Objective study or report**	**Main outcome / discussion point**
Baada et al. (24)	Yes	*Peer-Reviewed* Qualitative In-depth Interviews, FGDs	Ghana	Climate variability and change	Contraceptive use and family planning; autonomy; birth spacing; access to reproductive health services	Explore the reproductive health experience of women farmer's migrating as a result from climate variability	Migrant women face limited autonomy in family planning decisions, lack access to maternal and general health care services, and have limited social support in the migration destination.
Haque et al. (27)	Yes	*Peer-Reviewed* Quantitative Cross-Sectional Survey	Bangladesh	Flood and riverbank erosion	Maternal health services; utilisation of healthcare for delivery of post-natal care (PNC) for neonates; and trained provider-guided PNC services	Investigate the effect of displacement due to extreme climate events on whether a child is delivered at a health centre and on postnatal care service utilization	Climate-related displacement reduces mother's delivery and PNC service utilization. Displaced are more likely to have a home delivery.
Haque *et al*. (12)	Yes	*Peer-Reviewed* Quantitative Cross-Sectional Survey	Bangladesh	Flood and riverbank erosion	Maternal health services; antenatal care (ANC) visits; sources of ANC visits; local availability and accessibility of health care services	Examine the relationship between displacement resulting from extreme weather events and ANC service utilization.	Women from households that had been displaced three or more times were less likely than those from nondisplaced households to have received an ANC visit and at least four visits with a trained provider (OR 0.3 and 0.4 respectively).
Lindvall *et al*. (25)	No *Little on SRH*	*Peer-Reviewed* Qualitative In-depth interviews with validation workshop	Somalia, Kenya and Ethiopia	Drought	GBV; Maternal/child/ neonatal mortality	To identify knowledge regarding public health consequences of large-scale displacement from droughts or floods.	The treatment of GBV is insufficient, and IDPs have inadequate access to essential health services in refugee camps.
Pardhi *et al*. (26)	No *Little on climate change*	*Peer-Reviewed* Qualitative In-depth interviews	India	Drought	Maternal health; ANC utilisation	Examine sanitation, hygiene and living conditions of migrants forced to leave their homes because of the drought. Focus was on the health problems of pregnant migrant women and children in their destinations.	The study found that pregnant women did not have adequate sanitation, nutrition and rest. Pregnant women have missed important ANC care services.
D'souza (28)	No *Little on migration*	*Grey Literature* Blog Post	Asia-Pacific region	Sea-level rise Heatwaves	Risk of sexual violence and rape; reduced access to SRH services like contraceptives and ART; women, girls and LGBTQI groups included in analysis	Discussing the importance of including SRHR in climate change mitigation strategies.	The blog post outlines the need for multi-sectoral, multi-stakeholder approaches toward climate-related vulnerabilities, risk and gaps in accessing SRH Services for young people in the Asia-Pacific.
UNFPA (29)	Yes	*Grey Literature* Symposium report	African region	Droughts and other effects of climate change (broad)	GBV; rape; Transactional sex; child marriage; SRH service access	Focus on programmatic, partnership and financing opportunities linking ICPD, SRHR, gender and climate change resilience.	The key discussion points of this symposium centre around the intersectionality of vulnerabilities with regard to climate-related displacement and SRH in Africa and calls for more effective implementation of human right treaties.
IPPF (32)	No *Little on displacement*	*Grey Literature* Position paper	Global and/or general	Climate change (broad)	GBV; Child/early/forced marriage; Access to SRH & maternal health services	Description of IPPF's priorities for advocacy on the intersection of SRHR and the climate crisis.	The report gives a broad outline on the links between the climate crisis and SRH and the role of global health care providers as IPPF.
WHO (30)	No *Little on SRH*	*Grey Literature* Report	Global and/or general	Climate change (broad)	GBV; Harassment and loss of privacy in shelters and in relief queues	Providing a first overview on the interactions between gender, climate change and health.	This global report calls for research and work on climate change and health to be sensitive to gender dimensions of health care (including mental health) and health-seeking behaviours.
CARE (31)	Yes	*Grey Literature* Report	Global and/or general	Climate change (broad)	GBV; sexual violence at home: travelling further for water in drought; Child marriage; Increased sexual violence, transactional sex and trafficking	Outlining the consequences of climate-induced displacement and how the triple injustice of climate change, poverty and gender inequality must be met by transformative action.	The report calls for a gender-transformative and human-rights based way to tackle climate-induced displacement through protection of women's rights, female empowerment and funding directed to women.

*ANC, antenatal care; ART, antiretroviral treatment; FGD, focus group discussions; GBV, gender-based violence; ICPD, international conference on population and development; IDPs, internally displaced peoples; IPPF, International Planned Parenthood Federation; PNC, post-natal care; SRH, sexual and reproductive health; SRHR, sexual and reproductive health and rights; UNFPA, United Nations Population Fund; WHO, World Health Organization*.

### Migration Characteristics

In the reviewed literature, the concept of “climate migration” was applied to the range of migration contexts. Four studies acknowledged the SRH needs of displacement-prone or “trapped” populations at risk of migration due to climate factors (*n* = 4) (12, 27, 31, 33). Climate migration was recognised as a driver of forcible displacement and/or refugees, into camp settings or emergency shelters (*n* = 3), (25, 30, 31) and as a factor driving insidious migration, including seasonal and economic migration (*n* = 4), (12, 24, 27) and rural to urban migration (*n* = 2) 0.2 (5, 26).

### Sexual and Gender-Based Violence

Women migrating due to climate change were described as being vulnerable to different forms of SGBV including domestic and intimate partner violence, sexual violence (e.g. harassment or rape when attempting to receive relief services such as food or shelter), forced marriage and trafficking (29, 30). In Ethiopia, overcrowding at relief shelters as well as increased travel distances to reach fresh water increased women's risk of experiencing sexual violence (31). Adolescent girls in particular reported higher levels of sexual harassment and abuse as well as a lack of privacy in emergency shelters after natural disasters (30). This harassment and violence was also described within the home (e.g. increased risks of domestic and intimate partner violence) (30, 31). Even when the majority of migrants are men, for example in the flood-prone Kurigram District of Bangladesh, a by-product of migration was that girls and women may face sexual harassment in the absence of male household members – which again may result in forced marriage and stigmatisation (31). Furthermore, due to high vulnerability of some migrant women (e.g. those without documentation), coercion into transactional sex may occur (29, 31).

### Maternal and Neonatal Health

Access to and utilization of antenatal care (ANC) and postnatal care (PNC) were the main metrics reported in relation to maternal and neonatal health in the context of climate migration (12, 25–27, 32). Studies in Bangladesh reported that non-migrant women were more likely to receive ANC during their last pregnancy than women who had been displaced due to floods or riverbank erosion (12, 27). In a cross-sectional survey of 599 mothers, almost 60% of mothers from non-displaced households delivered at a health facility, whilst only 16% of displaced mothers experienced health facility deliveries (27). Likewise, a study in India found that women migrants forced to leave their home due to drought missed important ANC care services, including vaccinations and other treatments entitled under ANC (26). Furthermore, climate-displaced mothers were three times less likely to seek PNC for their neonates, especially from a trained provider (27). Those with a larger number of previous displacements had a higher extent of facility-based delivery and PNC service utilisation decrease. Higher maternal, child, and neonatal mortality rates were reported amongst displaced populations due to the lack of trained health care providers for these populations (25).

### Challenges and Barriers to SRH Services and Care

Included articles described a range of barriers faced by displaced women in accessing and utilising SRH services - from the lack of availability of such health services in new settings (24, 25, 27, 33) to financial and infrastructural challenges (24, 26, 27, 31). This included access to family planning services, contraception, and ART treatment for HIV (24, 29, 33). A study in India indicated that local government and administration, non-governmental organizations and local political parties failed to provide even basic sanitation facilities and maternal healthcare services to climate migrants (26). In patriarchal societies where women climate migrants did not have access to relief resources or control over allocation of resources at a household level, the ability to seek SRH was affected (31).

Forced migration and resettlement in a new community also affected individuals' knowledge of the new community's health systems and services and other support networks (24, 26). For example, women forced to migrate due to climate variability in the Upper West Region of Ghana described how the challenges they faced in traveling to and paying for health services were more difficult to overcome in their new communities where they did not have established relationships with neighbours and family members who could offer to help them (24). Other less-frequently listed barriers included a lack of women in organisational planning, (33) fears of stigma or violence, (32, 33) poor quality health services, (24) and implementation of services or interventions guided by donor priorities (25).

### Recommendations and Opportunities

Most commonly cited recommendations to address SRH in the context of climate migration included developing targeted health systems and services for women's SRH needs (*n* = 7), (12, 25–27, 29–31) inclusion and integration of local women into planning of services (*n* = 5), (24, 25, 30, 31, 33) and education/norm change around issues such as GBV (*n* = 6) (24, 25, 27, 29, 30, 33). Other recommendations and opportunities included economic support for women, (12, 24, 29, 30) gender-responsive adaptation and mitigation policies recognising climate migrants' SRH needs, (30) and improving referral pathways and resources for GBV (30). Strengthening the health systems of different communities and countries included incorporating wider ranges of care services (such as menstrual health and support for mental health services and GBV) (24, 30, 31) and improving infrastructure such as both physical and human resources (12).

A common theme across recommendations made in the documents was the involvement of all levels of stakeholders and actors, particularly affected women themselves, in the development and implementation of interventions (24, 25, 30, 31, 33). This was described at a variety of different levels from incorporating community-based interventions to change social norms (29) and emphasizing women-led aid that ensures women's participation in decision-making (31). An example of a gender-sensitive programme to build climate-resilience in Bolivia not only empowered women as decision-makers but also built longer-term capacity by incorporating their skills and expertise in the programme (30). Most importantly, there was a call for using existing community capacities and knowledge through community dialogues in the design and development of health care interventions. Local community health workers were identified as an avenue to connect between the health system and rural and remote communities (25).

## Discussion

To our knowledge, this scoping review provides the first systematically obtained and comprehensive overview of the evidence on climate migration and SRH. Most of the included documents discussed the relationship between climate migration and GBV (including child marriage and translational sex), maternal and neonatal health, accessibility and utilization of SRH services, and the challenges that inhibit receiving SRH care. This scoping review also identifies a number of recommendations addressing these challenges, such as targeting health systems strengthening and involving local communities.

A relevant consideration when discussing the influence of climate migration on SRH is whether the stressors on SRH result primarily from climate change-related factors, migration-induced factors, are additive when these two mediating factors meet, or whether there are unique stressors that emerge when these factors combine. For example, in the broader context of climate migration, the impacts on health, food and water security are important mediators for both mobility responses and health outcomes contextualised by climate change (1). A study in Bangladesh indicated that people displaced primarily due to climate change were more vulnerable to diseases than those displaced for other reasons (e.g. political), which was explained by inequalities in exposure to poor environmental conditions (1, 34, 35). While migration poses significant challenges and vulnerabilities affecting SRH, we expect climate-induced migration to come with additional specific challenges, such as climate-driven disruption of health services prior to migration, changes in baseline health and disease status - for example, vector-borne diseases, or malnutrition, and mental health stressors - and the failure to receive health services that recognise and meet these complex health vulnerabilities. Current evidence suggests that there are potentially important intersections between climate-migration-SRH, but further research can better assess this nexus.

The SRH outcomes reported in studies in this review focus on a relatively narrow range of SRH domains. The larger body of literature outlining the SRH impacts of climate change also include transmission and spread of STIs and other vector-borne diseases, as well as menstruation and malnutrition (36). Research exploring the SRH of migrants shows reduced use of screening and other services. The absence of these factors in this scoping review may well be due to the dearth of literature in this nexus, though it would be reasonable to extrapolate that the SRH impacts of both climate change and migration will be found in climate migrants, and that these impacts may be amplified due to compounding vulnerability. Different forms of climate migration are reflected in the literature - spanning at-risk or trapped populations, IDP's, refugees, displacement into camps and emergency shelters, rural-to-urban migration, and seasonal and economic migration. Responding to the SRH needs of climate migrants at different stages of migration requires migrant-sensitive health systems able to recognise, adapt, and respond to complex needs, particularly for hard-to-reach and at-risk populations (1). The absence of literature on this climate-migration-SRH nexus may arise from the this heterogeneity of need and vulnerability regarding the pathways between climate-migration and health, the lack of consensus on defining climate migration and its causal pathways, or as a result political and institutional deprioritization (1).

The population of focus in this review is women and girls, reflecting the language and emphasis of the majority of the literature base. However, it is important to note that climate migration may influence particular SRH risks for individuals with diverse gender identities, expressions, sexual orientations and sex characteristics (37). For example, gender and sexual minorities are often more severely impacted by natural disasters due to their frequent marginalization (14, 37). While none of the included documents explicitly addressed the SRH needs of men, access to family planning, contraception, and STI prevention are pertinent to men both within and outside of marriage, and men and boys are important to include in SRH interventions, programming and solutions (38, 39). Furthermore given climate-induced migration often involves the movement of men seeking employment, it is important to consider how this impacts them as well as their families' altered household structure (40). Achieving full and equitable SRH for the entire population, including in climate migration contexts, is not possible without engaging all genders and addressing gender inequality in societal and political structures (17).

Strategies recommended to address the health impacts of climate migration include pre-disaster planning, community consultation prior to resettlement, migrant-focused health schemes emphasizing access to health facilities, and coordination between the range of agency and government actors (41). In particular the importance of working with civil society organizations and relevant local actors/groups to develop, test, and tailor SRH interventions to their contexts has been emphasized (42, 43). Calls to engage communities in health have grown since the 1978 Alma Ata Declaration, resulting in the implementation of a number of community-based interventions that encourage community participation (44, 45). Involving communities, especially women and their families, beyond static educational interventions has become a key component in SRH interventions and services, especially family planning and maternal health programmes (45, 46). These include participatory community-based programmes such as women's groups and emergency transportation or care initiatives that provide support or delivery of services (47). Women's groups have become an increasingly implemented component to reproductive and maternal health programmes by empowering women as decision-makers in local communities through identifying and addressing local challenges and in their own health choices (48, 49). Examples in the literature on the participatory power of women in climate mitigation have pointed to their primary role as providers and users of energy, as climate activists, and as valuable perspectives of community interests (50–52). Bottom-up approaches, such as participatory women's groups, where SRH and climate change adaptation interventions are co-produced and delivered with relevant local actors are likely to be more feasible, acceptable and sustainable.

Our study has several strengths, including employing a detailed comprehensive search strategy to attempt to gather all available evidence, the synthesis of both peer-reviewed and grey literature, and a broad definition of SRH allowing for a diverse examination of SRH-related issues. It is also the first known study to examine SRH in the context of climate migration. However, there are several limitations. Firstly, the available evidence was limited and relatively low-quality, complicating robust information synthesis and preventing the performance of additional analyses (e.g. meta-analyses). Secondly, there is only a limited degree of integration of climate, meteorological and environment data in the individual studies that connect migration and SRH to climate change. Most studies refer to climate change in the narrative, or extrapolate from environmental events (e.g. floods, droughts) to climate change, but do not feature meteorological data demonstrating this relationship. This superficial linking of findings to climate change may risk compromising research quality, as do the assumptions linking climate change to migration where there is no consensus on causal pathways or definitions for climate migrants (1). An additional limitation of the data synthesized comes from respondent bias of the included participants. The women and girls affected were not always the respondents to questionnaires or surveys to collect data. For example, in several studies the heads of households, which are often men, or government and NGO representatives were the primary respondants (12, 25, 27). Finally, it is likely that the relationship between climate migration and SRH is heterogeneous across settings due to differences in exposure, vulnerability, sensitivity and adaptive capacity of migrating populations and host communities. This makes it unlikely for global findings to emerge and would suggest that local findings and recommendations are not generalizable to other settings. Consequently, forming overall conclusions on the interaction is challenging (1).

This scoping review highlights the need for an increase in the quantity and quality of empirical evidence on the effect of climate migration on SRH (the climate-migration-SRH nexus), as well as better characterisation and definitions between climate change and migration in the academic and operational literature, in order to design and implement climate-resilient, migrant-inclusive and gender-responsive health systems that can serve and meet the needs of these vulnerable populations. This research must be context-specific to reflect priorities and situational differences across localities and regions (53). Considerations need to be given to the degree in which meteorological data are meaningfully integrated into research exploring climate migration and SRH (1). Furthermore, there is an opportunity for this research to engage communities through locally led programming and for interventions to involve the affected populations through community representatives and participatory activities (52–55).

## Author Contributions

All authors have made substantial, intellectual contribution to the work, and approved it for publication.

## Funding

KD receives funding by the Gates Cambridge Scholarship (OPP1144) for her PhD research with publication fees paid by the Bill & Melinda Gates Foundations through KD's scholarship. Salary support for NS is provided under the RECAP project by the United Kingdom Research and Innovation as part of the Global Challenges Research Fund, grant number ES/P010873/1.

## Conflict of Interest

The authors declare that the research was conducted in the absence of any commercial or financial relationships that could be construed as a potential conflict of interest.

## Publisher's Note

All claims expressed in this article are solely those of the authors and do not necessarily represent those of their affiliated organizations, or those of the publisher, the editors and the reviewers. Any product that may be evaluated in this article, or claim that may be made by its manufacturer, is not guaranteed or endorsed by the publisher.
