# Supplementary material for: A Scoping Review to Assess Sexual and Reproductive Health Outcomes, Challenges and Recommendations in the Context of Climate Migration
Source: Front Glob Womens Health. 2021 Oct 15;2:757153. doi: 10.3389/fgwh.2021.757153 (PMC8594026; doi:10.3389/fgwh.2021.757153)
Supplement: Supplementary file 1 [file Data_Sheet_1.docx]

Supplementary Material

| **Supplement table 1 Search Strategy used on 2^nd^ July 2021** | |
| --- | --- |
| **Database** | **Terms** |
| **PubMed**  67 results | \| **Concept** \| **Terms** \| \| --- \| --- \| \| Sexual & Reproductive Health \| “reproductive health” OR “reproductive medicine” OR childbirth OR contracept* OR “fertility control” OR “fertility inhibition” OR “family plan” OR “family planning” OR “birth control” OR abortion* OR miscarriage* OR “sexual health” OR “sexually transmitted” OR STI* OR STD* OR “menstrual hygiene” OR “human immunodeficiency virus” OR HIV OR “prevention of mother-to-child transmission” OR PMTCT OR pregnan* OR “maternal health” OR “maternal welfare” OR antenatal OR prenatal OR postpartum OR perinatal OR postnatal OR ANC OR PNC OR AIDS OR “Acquired Immunodeficiency Syndrome” OR Peripartum OR “HPV” OR “human papilloma virus” OR “human papillomavirus” OR "GBV" OR “gender based violence” OR “gender-based violence” OR “sexual violence” OR “domestic violence” OR “emotional violence” OR "IPV" OR "FGM" OR "intimate partner violence" OR rape* OR "female genital mutilation" OR "genital cutting" OR "child marriage" OR "femicide" OR "fillicide"  OR  "Reproductive Health"[Mesh] OR "Reproductive Medicine"[Mesh] OR "Parturition"[Mesh] OR "Contraception"[Mesh] OR "Abortion, Induced"[Mesh] OR "Abortion, Spontaneous"[Mesh] OR "Sexual Health"[Mesh] OR "Sexually Transmitted Diseases"[Mesh] OR "HIV"[Mesh] OR "Acquired Immunodeficiency Syndrome"[Mesh] OR "Pregnancy"[Mesh] OR "Pregnant Women"[Mesh] OR "Maternal Health"[Mesh] OR "Maternal Welfare"[Mesh] OR "Prenatal Care"[Mesh] OR "Postnatal Care"[Mesh] OR "Postpartum Period"[Mesh] OR "Perinatal Care"[Mesh] OR "Peripartum Period"[Mesh] OR "Sex Offenses"[Mesh] or "Gender-Based Violence"[Mesh] or “battered women”[Mesh] \| \| Climate Change \| “climate change” OR “global warming” OR “climate variability” OR “climatic variability” OR “sea level rise” OR “greenhouse effect” OR “extreme weather” OR “environmental change” OR “climate disaster”  OR  "Climate Change"[Mesh] OR "Greenhouse Effect"[Mesh] OR "Extreme Weather"[Mesh] \| \| Migration \| migrat* OR immigrat* OR immigrant* OR emigrat* OR emigrant* OR migrant* OR outmigrat* OR refugee* OR “asylum seeker” OR “asylum seekers” OR “displaced person” OR “displaced persons” OR “displaced people” OR displacement OR diaspora* OR “population movement” OR “border crossing” OR “unaccompanied minor*” OR “climate migration” OR “climate induced migration”  OR  "Human Migration"[Mesh] OR "Transients and Migrants"[Mesh] OR "Emigrants and Immigrants"[Mesh] OR "Refugees"[Mesh] \| |
| **CINAHL**  26 results | \| **Concept** \| **Terms** \| \| --- \| --- \| \| Sexual & Reproductive Health \| “reproductive health” OR “reproductive medicine” OR childbirth OR contracept* OR “fertility control” OR “fertility inhibition” OR “family plan” OR “family planning” OR “birth control” OR abortion* OR miscarriage* OR “sexual health” OR “sexually transmitted” OR STI* OR STD* OR “menstrual hygiene” OR “human immunodeficiency virus” OR HIV OR “prevention of mother-to-child transmission” OR PMTCT OR pregnan* OR “maternal health” OR “maternal welfare” OR antenatal OR prenatal OR postpartum OR perinatal OR postnatal OR ANC OR PNC OR AIDS OR “Acquired Immunodeficiency Syndrome” OR Peripartum OR “HPV” OR “human papilloma virus” OR “human papillomavirus” OR "GBV" OR “gender based violence” OR “gender-based violence” OR “sexual violence” OR “domestic violence” OR “emotional violence” OR "IPV" OR "FGM" OR "intimate partner violence" OR rape* OR "female genital mutilation" OR "genital cutting" OR "child marriage" OR "femicide" OR "fillicide"  OR  *(MH "Reproductive Health") OR (MH "Family Planning") OR (MH "Maternal Health Services") OR (MH "Perinatal Care”) OR (MH “Childbirth”) OR (MH “Contraception”) OR (MH “Postnatal Period”) OR (MH “Prenatal Care”) OR (MH “Pregnancy”) OR (MH "Maternal Health Services") OR (MH "Sexual Health") OR (MH "Sexually Transmitted Diseases") OR (MH "HIV Infections+") OR (MH "Human Immunodeficiency Virus+") OR (MH "Gender-Based Violence") OR (MH "Domestic Violence") OR (MH "Dating Violence") OR (MH "Intimate Partner Violence")* \| \| Climate Change \| “climate change” OR “global warming” OR “climate variability” OR “climatic variability” OR “sea level rise” OR “greenhouse effect” OR “extreme weather” OR “environmental change” OR “climate disaster”  OR  *(MH "Climate Change") OR (MH "Greenhouse Effect") OR (MH "Extreme Weather”)* \| \| Migration \| migrat* OR immigrat* OR immigrant* OR emigrat* OR emigrant* OR migrant* OR outmigrat* OR refugee* OR “asylum seeker” OR “asylum seekers” OR “displaced person” OR “displaced persons” OR “displaced people” OR displacement OR diaspora* OR “population movement” OR “border crossing” OR “unaccompanied minor*” OR “climate migration” OR “climate induced migration”  OR  *(MH "Immigrants") OR (MH "Emigration and Immigration") OR (MH "Transients and Migrants") OR (MH "Refugees")* \| |
| **EMBASE**  317 results | \| **Concept** \| **Terms** \| \| --- \| --- \| \| Sexual & Reproductive Health \| “reproductive health” OR “reproductive medicine” OR childbirth OR contracept* OR “fertility control” OR “fertility inhibition” OR “family plan” OR “family planning” OR “birth control” OR abortion* OR miscarriage* OR “sexual health” OR “sexually transmitted” OR STI* OR STD* OR “menstrual hygiene” OR “human immunodeficiency virus” OR HIV OR “prevention of mother-to-child transmission” OR PMTCT OR pregnan* OR “maternal health” OR “maternal welfare” OR antenatal OR prenatal OR postpartum OR perinatal OR postnatal OR ANC OR PNC OR AIDS OR “Acquired Immunodeficiency Syndrome” OR Peripartum OR “HPV” OR “human papilloma virus” OR “human papillomavirus” OR "GBV" OR “gender based violence” OR “gender-based violence” OR “sexual violence” OR “domestic violence” OR “emotional violence” OR "IPV" OR "FGM" OR "intimate partner violence" OR rape* OR "female genital mutilation" OR "genital cutting" OR "child marriage" OR "femicide" OR "fillicide"  OR  *'reproductive health'/exp OR 'childbirth'/exp OR 'pregnancy'/exp OR 'birth control'/exp  OR 'abortion'/exp OR 'sexual health'/exp OR 'sexually transmitted disease'/exp OR 'mother to child transmission'/exp OR 'maternal care'/exp OR 'prenatal care'/exp OR 'puerperium'/exp OR 'postnatal care'/exp OR 'perinatal period'/exp OR 'Human immunodeficiency virus'/exp OR 'acquired immune deficiency syndrome'/exp OR 'gender based violence'/exp OR 'sexual violence'/exp OR 'domestic violence'/exp* \| \| Climate Change \| “climate change” OR “global warming” OR “climate variability” OR “climatic variability” OR  “sea level rise” OR “greenhouse effect” OR “extreme weather” OR “environmental change” OR “climate disaster”  OR  *'climate change'/exp OR 'greenhouse effect'/exp OR 'extreme weather'/exp OR 'environmental change'/exp* \| \| Migration \| migrat* OR immigrat* OR immigrant* OR emigrat* OR emigrant* OR migrant* OR outmigrat* OR refugee* OR “asylum seeker” OR “asylum seekers” OR “displaced person” OR “displaced persons” OR “displaced people” OR displacement OR diaspora* OR “population movement”  OR “border crossing” OR “unaccompanied minor*” OR “climate migration” OR “climate induced migration”  OR  *'migration'/exp OR 'migrant'/exp  OR 'refugee'/exp* \| |
| **Web of Science**  847 results | \| **Concept** \| **Terms** \| \| --- \| --- \| \| Sexual & Reproductive Health \| “reproductive health” OR “reproductive medicine” OR childbirth OR contracept* OR “fertility control” OR “fertility inhibition” OR “family plan” OR “family planning” OR “birth control” OR abortion* OR miscarriage* OR “sexual health” OR “sexually transmitted” OR STI* OR STD* OR “menstrual hygiene” OR “human immunodeficiency virus” OR HIV OR “prevention of mother-to-child transmission” OR PMTCT OR pregnan* OR “maternal health” OR “maternal welfare” OR antenatal OR prenatal OR postpartum OR perinatal OR postnatal OR ANC OR PNC OR AIDS OR “Acquired Immunodeficiency Syndrome” OR Peripartum OR “HPV” OR “human papilloma virus” OR “human papillomavirus” OR "GBV" OR “gender based violence” OR “gender-based violence” OR “sexual violence” OR “domestic violence” OR “emotional violence” OR "IPV" OR "FGM" OR "intimate partner violence" OR rape* OR "female genital mutilation" OR "genital cutting" OR "child marriage" OR "femicide" OR "fillicide" \| \| Climate Change \| “climate change” OR “global warming” OR “climate variability” OR “climatic variability” OR  “sea level rise” OR “greenhouse effect” OR “extreme weather” OR “environmental change” OR “climate disaster” \| \| Migration \| migrat* OR immigrat* OR immigrant* OR emigrat* OR emigrant* OR migrant* OR outmigrat* OR refugee* OR “asylum seeker” OR “asylum seekers” OR “displaced person” OR “displaced persons” OR “displaced people” OR displacement OR diaspora* OR “population movement”  OR “border crossing” OR “unaccompanied minor*” OR “climate migration” OR “climate induced migration” \| |
| **Scopus**  1132 results | \| **Concept** \| **Terms** \| \| --- \| --- \| \| Sexual & Reproductive Health \| “reproductive health” OR “reproductive medicine” OR childbirth OR contracept* OR “fertility control” OR “fertility inhibition” OR “family plan” OR “family planning” OR “birth control” OR abortion* OR miscarriage* OR “sexual health” OR “sexually transmitted” OR STI* OR STD* OR “menstrual hygiene” OR “human immunodeficiency virus” OR HIV OR “prevention of mother-to-child transmission” OR PMTCT OR pregnan* OR “maternal health” OR “maternal welfare” OR antenatal OR prenatal OR postpartum OR perinatal OR postnatal OR ANC OR PNC OR AIDS OR “Acquired Immunodeficiency Syndrome” OR Peripartum OR “HPV” OR “human papilloma virus” OR “human papillomavirus” OR "GBV" OR “gender based violence” OR “gender-based violence” OR “sexual violence” OR “domestic violence” OR “emotional violence” OR "IPV" OR "FGM" OR "intimate partner violence" OR rape* OR "female genital mutilation" OR "genital cutting" OR "child marriage" OR "femicide" OR "fillicide" \| \| Climate Change \| “climate change” OR “global warming” OR “climate variability” OR “climatic variability” OR  “sea level rise” OR “greenhouse effect” OR “extreme weather” OR “environmental change” OR “climate disaster” \| \| Migration \| migrat* OR immigrat* OR immigrant* OR emigrat* OR emigrant* OR migrant* OR outmigrat* OR refugee* OR “asylum seeker” OR “asylum seekers” OR “displaced person” OR “displaced persons” OR “displaced people” OR displacement OR diaspora* OR “population movement”  OR “border crossing” OR “unaccompanied minor*” OR “climate migration” OR “climate induced migration” \| |
| **Global Health**  447 results | \| **Concept** \| **Terms** \| \| --- \| --- \| \| Sexual & Reproductive Health \| “reproductive health” OR “reproductive medicine” OR childbirth OR contracept* OR “fertility control” OR “fertility inhibition” OR “family plan” OR “family planning” OR “birth control” OR abortion* OR miscarriage* OR “sexual health” OR “sexually transmitted” OR STI* OR STD* OR “menstrual hygiene” OR “human immunodeficiency virus” OR HIV OR “prevention of mother-to-child transmission” OR PMTCT OR pregnan* OR “maternal health” OR “maternal welfare” OR antenatal OR prenatal OR postpartum OR perinatal OR postnatal OR ANC OR PNC OR AIDS OR “Acquired Immunodeficiency Syndrome” OR Peripartum OR “HPV” OR “human papilloma virus” OR “human papillomavirus” OR "GBV" OR “gender based violence” OR “gender-based violence” OR “sexual violence” OR “domestic violence” OR “emotional violence” OR "IPV" OR "FGM" OR "intimate partner violence" OR rape* OR "female genital mutilation" OR "genital cutting" OR "child marriage" OR "femicide" OR "fillicide" \| \| Climate Change \| “climate change” OR “global warming” OR “climate variability” OR “climatic variability” OR  “sea level rise” OR “greenhouse effect” OR “extreme weather” OR “environmental change” OR “climate disaster” \| \| Migration \| migrat* OR immigrat* OR immigrant* OR emigrat* OR emigrant* OR migrant* OR outmigrat* OR refugee* OR “asylum seeker” OR “asylum seekers” OR “displaced person” OR “displaced persons” OR “displaced people” OR displacement OR diaspora* OR “population movement”  OR “border crossing” OR “unaccompanied minor*” OR “climate migration” OR “climate induced migration” \| |

**Supplement Table 2 Hand-searched websites (n=9) of organisations working on SRH and climate migration context**

| **Organisations** | **Website** |
| --- | --- |
| World Health Organization (WHO) | https://www.who.int/ |
| United Nations Children’s Emergency Fund (UNICEF) | https://www.unicef.org/ |
| International Planned Parenthood Federation (IPPF) | <https://www.ippf.org/> |
| Climate and migration | https://climatemigration.org.uk/ |
| United Nations High Commissioner for Refugees (UNHCR) | https://www.unhcr.org/ |
| International Organization for Migration (IOM) | https://www.iom.int/ |
| United Nations Relief and Works Agency (UNRWA) | <https://www.unrwa.org/> |
| International Migration Institute | <https://www.migrationinstitute.org/> |
| United Nations Population Fund (UNFPA) | <https://www.unfpa.org/> |

**Supplement Table 3 Critical appraisal of qualitative studies (n=3) using the Joanna Briggs Institute critical appraisal tool.**^22^

| **#** | **Checklist question** | **Baada *et al.* 2021**^24^ | **Lindvall *et al.* 2020**^25^ | **Pardhi *et al.* 2020**^26^ |
| --- | --- | --- | --- | --- |
| **1** | *Is there congruity between the stated philosophical perspective and the research methodology?* | Yes | Yes | Yes |
| **2** | *Is there congruity between the research methodology and the research question or objectives?* | Yes | Yes | Yes |
| **3** | *Is there congruity between the research methodology and the methods used to collect data?* | Yes | Yes | Yes |
| **4** | *Is there congruity between the research methodology and the representation and analysis of data?* | Yes | Yes | Yes |
| **5** | *Is there congruity between the research methodology and the interpretation of results?* | Yes | Yes | Yes |
| **6** | *Is there a statement locating the researcher culturally or theoretically?* | No | No | No |
| **7** | *Is the influence of the researcher on the research, and vice- versa, addressed?* | No | No | No |
| **8** | *Are participants, and their voices, adequately represented?* | Yes | No | Yes |
| **9** | *Is the research ethical according to current criteria or, for recent studies, and is there evidence of ethical approval by an appropriate body?* | Yes | No | Yes |
| **10** | *Were strategies to address incomplete follow up utilized?* | No | No | No |

**Supplement Table 4 Critical appraisal of cross-sectional studies (n=2) using the Joanna Briggs Institute critical appraisal tool.**^22^

| **#** | **Checklist question** | **Haque *et al.* 2020a**^27^ | **Haque *et al.* 2020b**^12^ |
| --- | --- | --- | --- |
| **1** | *Were the criteria for inclusion in the sample clearly defined?* | Yes | Yes |
| **2** | *Were the study subjects and the setting described in detail?* | Yes | No |
| **3** | *Was the exposure measured in a valid and reliable way?* | No | No |
| **4** | *Were objective, standard criteria used for measurement of the condition?* | No | No |
| **5** | *Were confounding factors identified?* | Unclear | Unclear |
| **6** | *Were strategies to deal with confounding factors stated?* | No | Unclear |
| **7** | *Were the outcomes measured in a valid and reliable way?* | No | No |
| **8** | *Was appropriate statistical analysis used?* | Yes | Yes |

**Supplement Table 5 Critical appraisal of the Grey Literature (n=5) using the Authority, Accuracy, Coverage, Objectivity, Date, Significance (AACODS) checklist.**^22^

| **#** | **Checklist question** | **UNFPA 2019^27^** | **IPPF 2021^30^** | **WHO 2014^28^** | **CARE 2020^29^** | **D'souza 2018^31^** |
| --- | --- | --- | --- | --- | --- | --- |
| 1 | *Authority – identifying who is responsible for the intellectual content* | Yes | Yes | Yes | Yes | Yes |
| 2 | *Accuracy – is there a stated aim or brief, methodology, peer-review, reputable and representative* | Yes | Yes | Yes | Yes | No |
| 3 | *Coverage – are the limits clearly stated* | Yes | Yes | Yes | Yes | Unclear |
| 4 | *Objectivity – is the authors standpoint clear? Does the work seem balanced?* | Yes | Yes | Yes | Yes | Unclear |
| 5 | *Date – is the item dated, relevant and contemporary* | Yes | Yes | Yes | Yes | Unclear |
| 6 | *Significance – is the item meaningful; does it add context?* | Yes | Yes | Yes | Yes | Unclear |

**Supplement Table 6 Preferred Reporting Items for Systematic Reviews and Meta-Analyses extension for Scoping Reviews (PRISMA-ScR) Checklist**

| **SECTION** | **#** | **PRISMA-ScR CHECKLIST ITEM** | **Page** |
| --- | --- | --- | --- |
| Title | 1 | Identify the report as a scoping review. | 1 |
| Structured summary | 2 | Provide a structured summary that includes (as applicable): background, objectives, eligibility criteria, sources of evidence, charting methods, results, and conclusions that relate to the review questions and objectives. | 2 |
| Rationale | 3 | Describe the rationale for the review in the context of what is already known. Explain why the review questions/objectives lend themselves to a scoping review approach. | 3 |
| Objectives | 4 | Provide an explicit statement of the questions and objectives being addressed with reference to their key elements (e.g., population or participants, concepts, and context) or other relevant key elements used to conceptualize the review questions and/or objectives. | 3 |
| Protocol and registration | 5 | Indicate whether a review protocol exists; state if and where it can be accessed (e.g., a Web address); and if available, provide registration information, including the registration number. | 4 |
| Eligibility criteria | 6 | Specify characteristics of the sources of evidence used as eligibility criteria (e.g., years considered, language, and publication status), and provide a rationale. | 4-5 |
| Information sources* | 7 | Describe all information sources in the search (e.g., databases with dates of coverage and contact with authors to identify additional sources), as well as the date the most recent search was executed. | 4-5 |
| Search | 8 | Present the full electronic search strategy for at least 1 database, including any limits used, such that it could be repeated. | Supplement table 1 |
| Selection of sources of evidence† | 9 | State the process for selecting sources of evidence (i.e., screening and eligibility) included in the scoping review. | 4-5 |
| Data charting process‡ | 10 | Describe the methods of charting data from the included sources of evidence (e.g., calibrated forms or forms that have been tested by the team before their use, and whether data charting was done independently or in duplicate) and any processes for obtaining and confirming data from investigators. | 4-5 |
| Data items | 11 | List and define all variables for which data were sought and any assumptions and simplifications made. | 4-5 |
| Critical appraisal of individual sources of evidence§ | 12 | If done, provide a rationale for conducting a critical appraisal of included sources of evidence; describe the methods used and how this information was used in any data synthesis (if appropriate). | 5 |
| Synthesis of results | 13 | Describe the methods of handling and summarizing the data that were charted. | 5 |
| Selection of sources of evidence | 14 | Give numbers of sources of evidence screened, assessed for eligibility, and included in the review, with reasons for exclusions at each stage, ideally using a flow diagram. | 5-6  16 Figure 1 |
| Characteristics of sources of evidence | 15 | For each source of evidence, present characteristics for which data were charted and provide the citations. | 5-9  17-18 Table 1 |
| Critical appraisal within sources of evidence | 16 | If done, present data on critical appraisal of included sources of evidence (see item 12). | 6  24-25 Supplement table 3-5 |
| Results of individual sources of evidence | 17 | For each included source of evidence, present the relevant data that were charted that relate to the review questions and objectives. | 5-9 |
| Synthesis of results | 18 | Summarize and/or present the charting results as they relate to the review questions and objectives. | 5-9 |
| Summary of evidence | 19 | Summarize the main results (including an overview of concepts, themes, and types of evidence available), link to the review questions and objectives, and consider the relevance to key groups. | 5-9 |
| Limitations | 20 | Discuss the limitations of the scoping review process. | 11 |
| Conclusions | 21 | Provide a general interpretation of the results with respect to the review questions and objectives, as well as potential implications and/or next steps. | 9-12 |
| Funding | 22 | Describe sources of funding for the included sources of evidence, as well as sources of funding for the scoping review. Describe the role of the funders of the scoping review. | 12 |

JBI = Joanna Briggs Institute; PRISMA-ScR = Preferred Reporting Items for Systematic reviews and Meta-Analyses extension for Scoping Reviews.

* Where *sources of evidence* (see second footnote) are compiled from, such as bibliographic databases, social media platforms, and Web sites.

† A more inclusive/heterogeneous term used to account for the different types of evidence or data sources (e.g., quantitative and/or qualitative research, expert opinion, and policy documents) that may be eligible in a scoping review as opposed to only studies. This is not to be confused with *information sources* (see first footnote).

‡ The frameworks by Arksey and O’Malley (6) and Levac and colleagues (7) and the JBI guidance (4, 5) refer to the process of data extraction in a scoping review as data charting*.*

§ The process of systematically examining research evidence to assess its validity, results, and relevance before using it to inform a decision. This term is used for items 12 and 19 instead of "risk of bias" (which is more applicable to systematic reviews of interventions) to include and acknowledge the various sources of evidence that may be used in a scoping review (e.g., quantitative and/or qualitative research, expert opinion, and policy document).
